# Appendicitis causing psoas and liver abscesses—a rare combination

**DOI:** 10.1093/jscr/rjag348

**Published:** 2026-05-06

**Authors:** Matea Dominkovic, George Chen

**Affiliations:** Department of General Surgery, Westmead Hospital, Westmead 2145, Australia; Department of General Surgery, Westmead Hospital, Westmead 2145, Australia

**Keywords:** appendicitis, liver abscess, psoas abscess

## Abstract

We present the case of a young, otherwise healthy male, who presented with synchronous psoas and liver abscesses secondary to acute appendicitis. These are both rare occurrences in the setting of acute appendicitis, which can dramatically increase the case complexity and mortality risk. Their occurrence synchronously has not previously been reported. The patient was managed acutely with percutaneous drainage of the liver abscess and long course antibiotic therapy before undergoing interval appendicectomy with colonoscopy.

## Introduction

Acute appendicitis is amongst the most common causes of acute abdominal pain presentations with surgery being the mainstay of treatment [1]. Appendiceal perforation can complicate the condition, and this can occur in 16%–40% of cases [1]. Perforated appendicitis increases the mortality from less than 0.1% to around 5% [1].

Liver abscess on the other hand is rare, but mortality approaches 15% [2]. More commonly pyogenic liver abscesses result from biliary infections that contaminate the biliary tract, however haematogenous spread through the portal vein can occur [2]. Of the 20% of liver abscesses secondary to non-biliary sources, only 1% of these were a complication of acute appendicitis [3]. It is more common in male patients with underlying immunosuppressive conditions such as diabetes or cancer [4].

Similarly, psoas abscess is also a rare condition occurring either due to haematogenous spread from distant sites or direct spread from nearby infection [5]. Historically tuberculosis of the spine was the most frequent cause, however gastrointestinal source is now more common, usually due to Crohn’s disease, but can also result from acute appendicitis [6]. Psoas abscess has a reported mortality of 2.4% in primary abscesses and up to 19% for secondary abscesses [7].

In this case, we report a case of both psoas abscess and liver abscesses complicating acute appendicitis, which despite the high reported mortality rates, was treated successfully with antibiotics, percutaneous drainage of liver abscesses and interval appendicectomy with colonoscopy. We aim to highlight the unusual presentation and combination of complications despite there being no identified risk factors, and we would advocate for colonoscopy to screen for malignancy.

## Case

A male in his late 30s presented to the Emergency Department with a 1-h history of severe right upper quadrant pain radiating to the right shoulder, with associated nausea. He denied fevers, rectal bleeding or change to bowel habits. His medical history included a bowel resection in childhood for an unspecified congenital abnormality. He was haemodynamically stable and afebrile. Examination revealed right upper quadrant tenderness with a negative Murphy’s sign. Blood tests demonstrated marked inflammatory response (white cell count 24.4 × 10^9^/l, neutrophilia; C-reactive protein 323 mg/l) and mildly deranged liver function tests.

**Figure 1:**
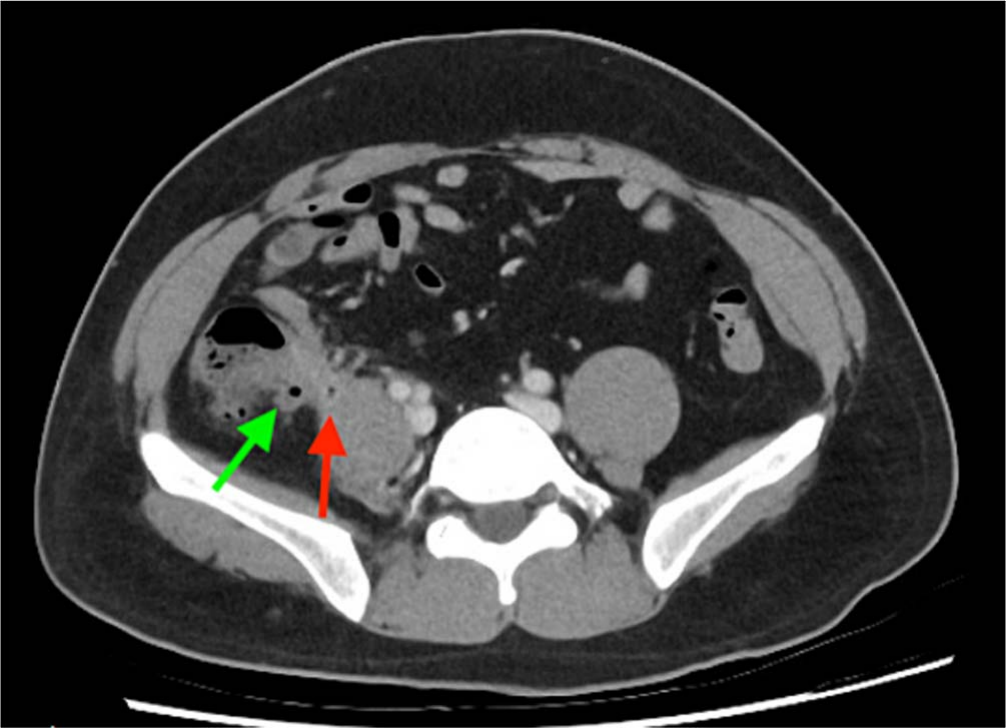
Inflamed appendix consistent with acute appendicitis (green arrow) with adjacent psoas abscess (red arrow).

**Figure 2:**
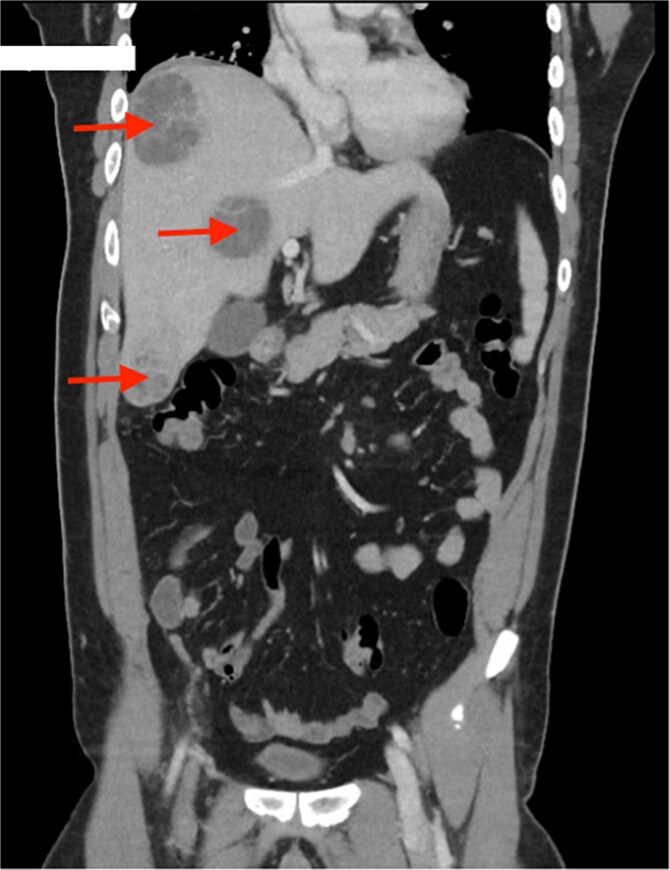
Multiple liver abscesses, the largest at segment 8 (red arrows).

Computed tomography (CT) scan revealed a thick-walled appendix of 14 mm in diameter with moderate surrounding fat stranding consistent with appendicitis and a small 10 mm adjacent right psoas abscess (Fig. 1). There were multiple lesions in the right lobe of the liver, the largest being in segment 8 with a diameter of 55 mm, representing abscesses (Fig. 2).

He was commenced on intravenous ceftriaxone and metronidazole, and on the third day of admission underwent percutaneous drainage of the largest segment 5 liver abscess. Following drainage, he continued to have fevers with elevated inflammatory markers, and minimal output from the drain. On Day 11 of admission, 2 further abscesses underwent percutaneous aspiration.

The drain fluid was sent for culture and revealed growth of *Streptococcus anginosus*. Blood tests for carcinoembryonic antigen, hydatid serology, and HIV screening were performed, which all returned negative. He subsequently improved clinically and biochemically and on Day 14 of admission his drain was removed, and he was discharged on oral antibiotics, Augmentin Duo Forte.

On follow-up in clinics, progress CT abdomen pelvis scans were performed at 2 weeks, 6 weeks, and 4 months post-discharge, which demonstrated gradual improvement then resolution of the liver abscesses, but persistent tethering of the appendix to the psoas. He remained asymptomatic and his oral antibiotics were ceased after an 8-week course.

At 6 months, he presented for elective laparoscopic appendicectomy and colonoscopy. His colonoscopy was unremarkable. His laparoscopic procedure was complicated by dense adhesions to the anterior abdominal wall at the site of the scar from his previously surgery which require adhesiolysis. There was also dense fibrosis of the appendix to the retroperitoneum and a stapled caecectomy had to be performed. He had an unremarkable recover postoperatively. The histopathology analysis of his appendix specimen demonstrated no evidence of dysplasia or malignancy.

## Discussion

Appendicitis is a common presentation, but psoas abscess and liver abscess individually are both rare entities, even more rare as a complication of appendicitis. To the author’s knowledge, this is the first published case report of a patient with both psoas abscess and multiple liver abscesses complicating acute appendicitis.

Commonly, appendicitis is a polymicrobial infection, however the most common bacterium seen are *Escherichia Coli*, Streptococcus species, *Pseudomonas aeruginosa*, and Bacteroides species [8]. Bacterial infections are the most common cause of pyogenic liver abscess, and nearly half of these are also polymicrobial with *Streptococcus milleri*, Klebsiella species and anaerobes being most commonly identified [9]. *Streptococcus anginosus* belongs to the *Streptococcus milleri* group of bacteria and these are usually opportunistic in nature [10]. It is usually seen in patients with multiple comorbidities, cancer, and diabetes [9]. The development of multiple liver abscesses in an immunocompetent patient is uncommon [10].

Additionally, a significantly higher incidence of colorectal cancer has been seen up to 3 years after pyogenic liver abscess diagnosis and a high index of suspicion is needed [11]. Therefore, studies have found that offering screening for colorectal cancer to these patients may be useful, which is why colonoscopy was performed in this case [11].

Appendicitis management remains surgical [1] but imaging-guided percutaneous drainage is the first-line management of pyogenic liver abscess [12]. Percutaneous drainage also allows for the culprit organism to be identified to guide antibiotic therapy as blood cultures often are not reflective in liver abscess [12]. Duration of therapy for liver abscess has not been determined by randomized control trials; however, treatment generally varies from 2 to 6 weeks, and drainage is important [13].

Our patient was not immunosuppressed and did not have any other risk factors for these complications, and this case highlights the importance of maintaining a level of suspicion as appendicitis has potential to cause complication. Additionally, it is important to remain vigilant for underlying malignancy and tumour markers and colonoscopy was performed in this case.

## Conclusion

We highlight a case of an otherwise healthy male with psoas abscess and liver abscesses resulting from appendicitis. It highlights the successful management of multiple synchronous complications of acute appendicitis and emphasizes the importance of maintaining vigilance even in otherwise healthy patients. We recommend screening for risk factors like HIV and considering the risk of colorectal malignancy.
